# Supplementary material for: Lack of TLR4 modifies the miRNAs profile and attenuates inflammatory signaling pathways
Source: PLoS One. 2020 Aug 11;15(8):e0237066. doi: 10.1371/journal.pone.0237066 (PMC7418977; doi:10.1371/journal.pone.0237066)
Supplement: S1 Table — (PDF) [file pone.0237066.s001.pdf]

Supplementary Table 1S: List of miRNAs counts for each condition studied.

miRNA	WT1	WT2	WT3	KO2	KO3
mmu-miR-1982-3p	0	0	0	2	2
mmu-miR-5122	0	0	0	2	2
mmu-miR-5114	0	0	0	2	3
mmu-miR-677-3p	0	0	0	3	2
mmu-let-7c-1-3p	0	2	2	0	0
mmu-miR-135b-5p	0	2	2	0	0
mmu-miR-218-2-3p	2	2	0	0	0
mmu-miR-296-3p	2	0	2	0	0
mmu-miR-322-3p	2	2	0	0	0
mmu-miR-7019-3p	2	2	0	0	0
mmu-miR-7066-3p	2	0	2	0	0
mmu-miR-140-5p	2	0	2	0	2
mmu-miR-144-5p	2	3	0	0	0
mmu-miR-187-5p	3	2	0	0	0
mmu-miR-1947-5p	3	2	0	0	0
mmu-miR-222-5p	3	2	0	0	0
mmu-miR-299a-3p	2	3	0	0	0
mmu-miR-30d-3p	0	2	3	0	0
mmu-miR-376c-3p	3	0	2	0	0
mmu-miR-669a-3p	0	2	3	0	0
mmu-miR-153-5p	0	2	3	0	2
mmu-miR-25-5p	0	3	2	2	0
mmu-miR-92b-5p	0	3	2	3	0
mmu-miR-6240	0	3	2	6	0
mmu-miR-677-5p	2	3	0	4	3
mmu-let-7a-1-3p	2	2	2	0	0
mmu-miR-376a-3p	2	2	2	0	0
mmu-miR-486a-3p	4	2	0	0	0
mmu-miR-539-5p	0	4	2	0	0
mmu-miR-20a-5p	3	0	3	0	2
mmu-miR-3061-5p	4	2	0	2	0
mmu-miR-7015-3p	3	0	3	2	0
mmu-miR-137-5p	2	2	2	4	0
mmu-miR-1943-5p	3	3	0	5	0
mmu-miR-210-5p	0	4	2	0	5
mmu-let-7g-3p	0	5	2	0	0
mmu-miR-191-3p	0	4	3	0	0
mmu-miR-195a-3p	0	5	2	0	0
mmu-miR-204-3p	2	0	5	0	0
mmu-miR-3084-3p	3	2	2	0	0
nmu-miR-3102-3p.2-3	2	5	0	0	0
mmu-miR-679-5p	4	3	0	0	0
mmu-miR-7068-3p	4	3	0	0	0
mmu-miR-12191-3p	3	2	2	0	2
mmu-miR-29a-5p	2	3	2	2	0
mmu-miR-764-5p	0	4	3	3	0
mmu-miR-183-3p	2	4	2	0	0
mmu-miR-211-5p	0	6	2	0	0

mmu-miR-3083b-3p	2	3	3	0	0
mu-miR-3102-5p.2-5	3	2	3	0	0
mmu-miR-344d-3-5p	2	4	2	0	0
mmu-miR-374b-5p	2	6	0	0	0
mmu-miR-665-3p	3	0	5	0	0
mmu-miR-7068-5p	3	5	0	0	0
mmu-miR-325-5p	2	4	2	2	0
mmu-miR-7046-3p	3	3	2	2	0
mmu-miR-99a-3p	0	5	3	3	0
mmu-miR-1247-5p	2	4	2	3	4
mmu-miR-1964-3p	3	2	3	5	3
mmu-miR-412-5p	2	3	3	7	7
mmu-miR-3061-3p	4	2	3	0	0
mmu-miR-3083-5p	4	3	2	0	0
mmu-miR-6516-5p	6	3	0	0	0
mmu-miR-879-5p	5	2	2	2	0
mmu-miR-145a-3p	4	3	3	0	0
mmu-miR-19b-3p	3	4	3	0	0
mmu-miR-7226-3p	4	6	0	0	0
mmu-miR-412-3p	4	4	2	0	2
mmu-miR-29b-2-5p	4	2	4	5	0
mmu-miR-93-3p	0	10	0	2	3
mmu-miR-1264-3p	4	4	3	0	0
mmu-miR-671-5p	5	0	6	0	0
mmu-miR-3082-3p	4	4	3	2	0
mmu-miR-7224-3p	0	5	6	2	2
mmu-miR-3057-5p	2	5	4	8	0
mmu-miR-134-3p	0	7	4	4	6
mmu-miR-350-3p	0	7	5	0	0
mmu-miR-1306-5p	4	4	4	3	0
mmu-miR-34c-3p	4	3	5	3	0
mmu-miR-669c-5p	6	2	4	2	2
mmu-miR-138-2-3p	6	4	2	5	2
mmu-miR-345-3p	2	7	4	0	0
mmu-miR-148b-5p	5	5	3	0	2
mmu-miR-5129-3p	5	4	4	6	0
mmu-miR-500-3p	3	5	5	4	3
mmu-miR-199a-3p	5	6	3	0	0
mmu-miR-96-5p	3	4	7	0	0
mmu-miR-7b-5p	4	7	3	2	0
mmu-miR-218-1-3p	4	4	6	8	3
mmu-miR-3086-5p	5	5	5	2	0
mmu-miR-1983	7	2	6	2	2
mmu-miR-543-5p	3	4	8	4	0
mmu-miR-323-5p	5	4	6	2	3
mmu-miR-540-5p	4	6	5	2	3
mmu-miR-17-5p	4	11	0	6	3
mmu-miR-199b-3p	4	5	7	2	0
mmu-miR-99b-3p	8	3	5	2	2
mmu-miR-34b-3p	5	7	4	3	2

mmu-miR-28a-3p	2	8	6	4	3
mmu-miR-3547-3p	2	9	5	5	3
mmu-miR-764-3p	7	5	5	3	0
mmu-miR-29c-5p	7	6	4	5	0
mmu-miR-322-5p	5	7	5	2	3
mmu-miR-342-5p	8	5	4	4	4
mmu-miR-8114	5	7	5	10	8
mmu-miR-27b-5p	3	10	5	0	0
mmu-miR-200a-5p	4	9	5	3	0
mmu-miR-504-5p	7	6	5	3	0
mmu-miR-130b-3p	7	3	8	4	0
mmu-miR-325-3p	5	7	6	4	0
mmu-miR-344b-3p	7	5	6	0	4
mmu-miR-551b-3p	2	10	6	5	0
mmu-miR-377-3p	7	9	3	0	0
mmu-miR-382-3p	6	2	11	0	0
mmu-miR-350-5p	9	8	2	2	3
mmu-miR-139-3p	9	6	4	3	6
mmu-miR-3072-3p	5	5	9	3	6
mmu-miR-30c-1-3p	10	7	3	8	3
mmu-let-7b-3p	8	9	4	0	2
mmu-miR-135a-5p	5	10	6	4	0
mmu-miR-7689-3p	6	6	9	4	2
mmu-miR-384-3p	6	11	5	2	0
mmu-miR-3085-3p	5	8	9	3	4
mmu-miR-339-3p	8	10	4	2	5
mmu-miR-200b-5p	3	12	8	2	0
mmu-miR-148a-5p	7	11	5	2	5
mmu-miR-409-5p	9	11	3	6	6
mmu-miR-582-5p	7	9	8	3	2
mmu-miR-700-3p	3	13	8	4	2
mmu-miR-1843a-3p	5	9	10	6	2
mmu-miR-125a-3p	10	7	7	8	5
mmu-miR-3552	5	11	9	6	6
mmu-miR-592-5p	7	10	9	0	0
mmu-miR-106b-5p	8	8	10	3	2
mmu-miR-3475-3p	9	9	8	4	3
mmu-miR-299a-5p	9	9	8	5	3
mmu-miR-3093-5p	8	12	6	7	3
mmu-miR-672-5p	9	11	6	6	4
mmu-miR-7047-3p	10	6	10	6	4
mmu-miR-378c	10	10	6	8	4
mmu-miR-702-3p	9	10	7	13	5
mmu-miR-6540-5p	4	13	10	6	4
mmu-miR-7a-2-3p	7	12	8	2	8
mmu-let-7i-3p	6	16	5	8	6
mmu-miR-760-3p	13	9	5	7	14
mmu-miR-376b-3p	6	15	7	2	0
mmu-miR-7a-5p	9	9	10	4	6
mmu-miR-3078-5p	8	15	5	8	5

mmu-miR-7080-3p	4	19	5	6	9
mmu-miR-7a-1-3p	5	10	14	0	0
mmu-miR-130b-5p	6	14	9	4	0
mmu-miR-877-5p	6	11	12	7	5
mmu-miR-877-3p	8	14	7	15	11
mmu-miR-210-3p	17	5	8	5	2
mmu-miR-128-2-5p	10	10	10	6	9
mmu-miR-455-3p	5	22	3	15	10
mmu-miR-433-5p	7	15	9	5	4
mmu-miR-378a-5p	9	12	10	6	5
mmu-miR-744-3p	14	8	10	6	2
mmu-miR-770-5p	11	11	10	11	3
mmu-miR-22-5p	13	7	13	2	2
mmu-let-7j	16	6	11	6	0
mmu-miR-203-3p	13	8	13	2	2
mmu-miR-574-3p	11	16	8	8	6
mmu-miR-137-3p	15	12	9	0	6
mmu-miR-340-3p	11	15	10	7	2
mmu-miR-376a-5p	10	14	13	2	2
mmu-miR-28a-5p	10	17	10	2	3
mmu-miR-488-5p	11	12	14	4	7
mmu-miR-1839-3p	6	22	10	2	3
mmu-miR-184-3p	14	14	10	5	2
mmu-miR-425-3p	11	19	8	6	8
mmu-miR-301a-3p	3	16	20	6	3
mmu-miR-296-5p	9	18	13	8	9
mmu-miR-138-1-3p	10	16	15	10	5
mmu-miR-3068-3p	11	15	17	5	5
mmu-miR-15b-5p	15	18	10	8	9
mmu-miR-324-3p	17	9	19	9	4
mmu-miR-153-3p	9	13	24	7	8
mmu-miR-185-5p	18	17	11	7	8
mmu-miR-1198-5p	18	18	10	11	7
mmu-miR-124-5p	13	17	16	8	10
mmu-miR-194-5p	13	21	13	4	7
mmu-miR-700-5p	12	16	21	9	5
mmu-miR-488-3p	14	9	29	4	2
mmu-miR-874-5p	11	19	23	6	6
mmu-miR-29b-3p	14	21	18	5	8
mmu-miR-365-3p	15	19	19	10	4
mmu-miR-501-3p	17	20	16	14	7
mmu-miR-134-5p	18	16	19	14	8
mmu-miR-3099-3p	13	32	9	12	6
mmu-miR-674-5p	24	13	18	9	7
mmu-let-7e-3p	16	20	20	12	4
mmu-miR-1981-3p	21	24	11	16	9
mmu-miR-377-5p	23	14	19	22	10
mmu-miR-770-3p	21	19	17	20	9
mmu-miR-1981-5p	17	25	15	22	11
mmu-miR-676-5p	22	22	16	8	5

mmu-miR-532-3p	17	25	18	15	13
mmu-miR-1224-5p	20	23	17	19	18
mmu-miR-872-5p	17	18	26	2	2
mmu-miR-339-5p	18	24	19	18	10
mmu-miR-24-2-5p	27	14	22	9	7
mmu-miR-369-3p	17	22	25	6	3
mmu-miR-106b-3p	21	24	20	6	8
mmu-miR-329-3p	22	25	18	11	8
mmu-miR-34a-5p	38	11	16	10	13
mmu-miR-345-5p	18	32	19	9	3
mmu-miR-664-5p	20	26	24	25	17
mmu-miR-379-3p	17	26	28	3	3
mmu-miR-30c-2-3p	31	19	24	16	10
mmu-miR-346-5p	27	23	28	24	16
mmu-miR-221-5p	21	27	31	9	8
mmu-miR-329-5p	33	24	25	17	7
mmu-miR-652-3p	28	32	22	23	5
mmu-miR-496a-3p	25	28	30	4	3
mmu-miR-344-3p	33	26	24	14	9
mmu-miR-935	19	39	27	27	19
mmu-miR-148b-3p	21	32	33	9	2
mmu-miR-15a-5p	30	28	32	7	11
mmu-miR-376b-5p	29	31	30	5	13
mmu-miR-154-5p	34	28	30	16	11
mmu-miR-379-5p	28	32	33	12	6
mmu-miR-324-5p	32	32	32	24	14
mmu-miR-673-3p	34	29	33	21	19
mmu-miR-6944-3p	15	55	26	31	30
mmu-miR-146b-5p	50	17	32	9	7
mmu-miR-146a-5p	32	34	33	9	8
mmu-miR-421-3p	36	41	24	16	7
mmu-miR-125b-1-3p	27	46	28	20	23
mmu-miR-708-5p	32	38	32	15	13
mmu-miR-370-5p	46	25	31	17	12
mmu-miR-384-5p	36	44	27	13	9
mmu-miR-335-5p	31	42	35	7	2
mmu-miR-873a-3p	33	39	36	24	17
mmu-miR-495-3p	34	31	46	6	11
mmu-miR-195a-5p	44	36	31	12	8
mmu-miR-540-3p	52	28	31	25	19
mmu-miR-666-3p	36	43	34	30	11
mmu-miR-598-3p	36	33	45	12	7
mmu-miR-200c-3p	17	60	41	5	0
mmu-miR-142a-5p	40	45	33	7	10
mmu-miR-10a-5p	35	57	26	18	15
mmu-miR-3059-5p	42	51	26	18	16
mmu-miR-673-5p	44	40	35	41	16
mmu-miR-497a-5p	35	49	36	25	10
mmu-miR-101b-3p	35	37	51	6	13
mmu-miR-431-5p	39	47	38	24	9

mmu-miR-338-5p	40	44	41	13	11
mmu-miR-133a-3p	39	47	46	26	22
mmu-miR-666-5p	28	61	45	34	25
mmu-miR-34c-5p	63	33	39	19	24
mmu-miR-9-3p	45	59	32	11	13
mmu-miR-758-3p	30	62	45	21	25
mmu-miR-3102-3p	42	55	40	42	13
mmu-miR-375-3p	44	54	40	17	6
mmu-miR-136-5p	49	47	42	15	16
mmu-miR-874-3p	52	46	40	37	25
mmu-miR-708-3p	54	50	36	11	10
mmu-miR-98-5p	51	42	48	22	19
mmu-miR-1839-5p	55	45	42	18	14
mmu-miR-380-3p	42	54	52	24	10
mmu-miR-543-3p	62	43	45	21	19
mmu-miR-490-3p	46	63	42	33	19
mmu-miR-873a-5p	51	46	56	21	22
mmu-miR-130a-3p	45	59	50	12	12
mmu-miR-31-5p	64	38	56	40	27
mmu-miR-1298-5p	43	51	65	21	22
mmu-miR-674-3p	52	58	52	38	37
mmu-miR-423-5p	64	53	45	53	39
mmu-miR-323-3p	51	72	43	28	18
mmu-miR-411-3p	56	53	60	16	18
mmu-miR-27a-3p	62	55	54	22	21
mmu-miR-1298-3p	43	75	55	18	7
mmu-miR-141-3p	20	99	56	2	0
mmu-miR-193b-3p	47	62	68	49	31
mmu-miR-29c-3p	48	84	53	10	14
mmu-miR-382-5p	60	66	59	31	16
mmu-miR-344d-3p	55	71	60	37	29
mmu-miR-10b-5p	81	54	53	7	15
mmu-miR-181a-2-3p	65	77	47	42	39
mmu-miR-187-3p	39	85	67	21	11
mmu-miR-370-3p	71	65	58	43	19
mmu-miR-872-3p	57	90	49	34	26
mmu-miR-671-3p	73	70	54	54	38
mmu-miR-181c-3p	91	63	53	32	25
mmu-miR-340-5p	62	63	83	19	17
mmu-miR-335-3p	60	69	80	19	8
mmu-miR-330-5p	71	67	74	45	33
mmu-miR-21a-5p	76	76	68	9	23
mmu-miR-1843b-3p	67	89	70	71	52
mmu-miR-337-5p	75	85	69	36	23
mmu-miR-330-3p	63	85	81	78	55
mmu-miR-451a	85	91	55	9	9
mmu-miR-361-5p	81	78	74	41	29
mmu-miR-331-3p	59	79	95	56	50
mmu-miR-425-5p	70	102	64	38	19
mmu-miR-485-3p	65	103	69	75	43

mmu-miR-326-3p	78	75	88	77	25
mmu-miR-127-5p	83	83	81	23	29
mmu-miR-320-3p	90	94	73	68	50
mmu-miR-125b-2-3p	97	77	87	34	26
mmu-miR-423-3p	86	105	72	59	51
mmu-miR-361-3p	100	93	70	58	60
mmu-miR-129-1-3p	78	109	77	88	58
mmu-miR-181d-5p	101	93	74	31	21
mmu-miR-93-5p	85	86	99	25	27
mmu-miR-532-5p	100	95	79	37	27
mmu-miR-1843b-5p	114	78	88	50	37
mmu-miR-1843a-5p	114	78	92	31	28
mmu-miR-487b-3p	84	116	88	24	24
mmu-miR-485-5p	96	111	89	96	64
mmu-miR-298-5p	78	137	87	74	38
mmu-miR-351-5p	127	93	82	79	67
mmu-miR-431-3p	106	99	98	79	47
mmu-miR-664-3p	98	123	96	94	72
mmu-miR-25-3p	98	128	94	19	21
mmu-miR-1291	82	153	92	111	71
mmu-miR-212-5p	114	107	110	72	39
mmu-miR-192-5p	137	114	100	29	34
mmu-miR-132-5p	135	108	118	53	39
mmu-miR-145a-5p	108	145	111	91	68
mmu-miR-101a-3p	117	113	138	29	28
mmu-miR-30e-3p	116	135	120	34	40
mmu-miR-218-5p	132	103	139	35	28
mmu-miR-126a-3p	121	124	131	27	29
mmu-miR-676-3p	117	147	112	81	68
mmu-miR-30a-3p	148	144	133	57	40
mmu-miR-667-3p	91	199	136	102	110
mmu-miR-219a-2-3p	170	135	127	46	45
mmu-miR-128-1-5p	201	110	129	137	103
mmu-miR-23a-3p	163	131	149	70	34
mmu-miR-181a-1-3p	166	162	116	70	85
mmu-miR-200a-3p	58	240	148	3	0
mmu-miR-338-3p	119	187	153	42	43
mmu-miR-582-3p	153	153	157	50	42
mmu-miR-369-5p	162	181	121	69	62
mmu-miR-200b-3p	79	253	136	12	0
mmu-miR-24-3p	189	122	160	71	56
mmu-miR-107-3p	177	164	141	67	49
mmu-miR-126a-5p	149	168	187	50	51
mmu-miR-26b-5p	160	189	162	30	34
mmu-miR-668-3p	163	207	164	124	113
mmu-miR-484	139	218	188	129	90
mmu-miR-341-3p	206	152	192	74	57
mmu-miR-212-3p	183	177	192	139	87
mmu-miR-99a-5p	202	202	150	86	89
mmu-miR-148a-3p	179	206	185	57	41

mmu-miR-433-3p	180	231	195	185	170
mmu-miR-1249-3p	200	260	175	186	180
mmu-miR-23b-3p	231	210	211	112	76
mmu-miR-136-3p	218	232	274	46	45
mmu-miR-429-3p	102	466	234	10	3
mmu-miR-140-3p	291	299	252	179	117
mmu-let-7d-3p	245	335	313	247	151
mmu-miR-92a-3p	270	356	268	214	146
mmu-miR-409-3p	346	347	286	163	159
mmu-miR-383-5p	431	282	358	176	110
mmu-miR-434-5p	374	357	340	160	131
mmu-miR-30b-5p	325	424	370	100	80
mmu-miR-129-5p	396	399	434	242	167
mmu-miR-139-5p	386	438	437	289	158
mmu-miR-300-3p	416	465	430	229	156
mmu-miR-100-5p	435	521	380	222	170
mmu-miR-221-3p	521	377	451	161	116
mmu-miR-328-3p	445	518	432	450	346
mmu-miR-342-3p	486	510	418	342	245
mmu-miR-150-5p	401	621	413	338	210
mmu-miR-378a-3p	495	466	479	233	165
mmu-miR-149-5p	429	581	485	351	249
mmu-miR-744-5p	514	541	492	432	347
mmu-miR-5121	432	653	496	591	340
mmu-miR-129-2-3p	503	619	538	543	353
mmu-let-7e-5p	648	512	513	264	205
mmu-miR-186-5p	519	636	538	185	151
mmu-miR-3535	483	705	560	515	331
mmu-let-7i-5p	724	542	603	365	216
mmu-let-7d-5p	854	548	718	367	272
mmu-miR-222-3p	709	773	643	437	369
mmu-miR-103-3p	943	736	725	342	264
mmu-miR-30e-5p	802	817	796	284	286
mmu-let-7g-5p	979	645	794	403	306
mmu-miR-151-5p	847	887	766	422	324
mmu-miR-411-5p	925	789	902	273	204
mmu-miR-381-3p	929	856	841	340	260
mmu-miR-16-5p	881	1026	837	273	204
mmu-let-7b-5p	1199	939	922	771	486
mmu-miR-204-5p	929	1365	854	417	417
mmu-miR-183-5p	505	1653	1111	53	19
mmu-miR-151-3p	957	1330	1022	867	705
mmu-miR-124-3p	1325	1189	1082	707	519
mmu-miR-541-5p	1480	1027	1110	406	370
mmu-miR-181b-5p	1411	1145	1098	515	430
mmu-miR-486b-5p	1434	1398	1093	789	885
mmu-miR-486a-5p	1448	1329	1170	795	983
mmu-miR-143-3p	1376	1435	1488	416	328
mmu-miR-138-5p	1178	1731	1683	614	399
mmu-let-7f-5p	1740	1396	1551	533	491

mmu-miR-181c-5p	2055	1543	1511	815	658
mmu-miR-132-3p	2368	1762	1902	1638	858
mmu-miR-29a-3p	1758	2418	1887	849	481
mmu-miR-410-3p	1992	2078	2139	1240	734
mmu-miR-27b-3p	2323	1882	2132	718	548
mmu-let-7a-5p	2855	1913	2402	1123	777
mmu-miR-30c-5p	2274	2870	2248	992	753
mmu-miR-9-5p	2489	2323	2686	644	540
mmu-miR-92b-3p	2363	3436	2486	2569	2303
mmu-miR-30a-5p	3692	2736	3270	1210	991
mmu-let-7c-5p	4138	2904	3480	1986	1348
mmu-miR-125b-5p	3727	4171	3134	3294	2627
mmu-miR-99b-5p	3608	4448	3894	4366	3035
mmu-miR-191-5p	4412	4430	3544	2681	2050
mmu-miR-182-5p	2159	6187	4145	185	65
mmu-miR-30d-5p	6638	5480	4965	3492	2712
mmu-miR-125a-5p	6623	8369	6225	6839	5215
mmu-miR-22-3p	7828	7667	7351	2884	2123
mmu-miR-128-3p	7402	8562	7184	3100	2203
mmu-miR-434-3p	11786	10441	9679	5112	4378
mmu-miR-26a-5p	11678	13614	10697	4324	3183
mmu-miR-127-3p	31124	34608	30933	18534	12848
mmu-miR-181a-5p	56753	37175	39861	28017	20451
