# Supplementary figures and images for: Lack of TLR4 modifies the miRNAs profile and attenuates inflammatory signaling pathways
Source: PLoS One. 2020 Aug 11;15(8):e0237066. doi: 10.1371/journal.pone.0237066 (PMC7418977; doi:10.1371/journal.pone.0237066)

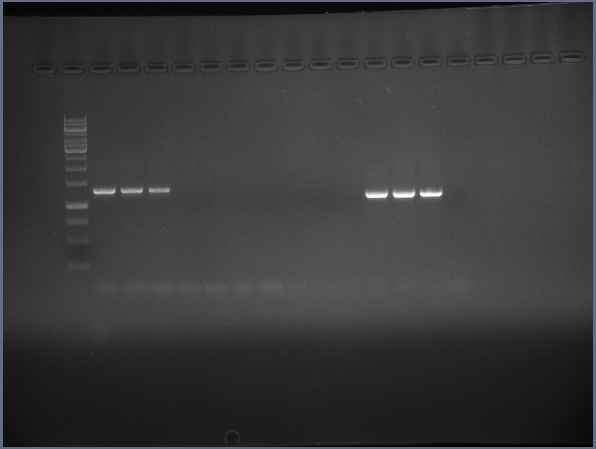

Supplement: S1 Raw images — (TIF) [file pone.0237066.s002.tif]
