# Supplementary material for: Lack of TLR4 modifies the miRNAs profile and attenuates inflammatory signaling pathways
Source: PLoS One. 2020 Aug 11;15(8):e0237066. doi: 10.1371/journal.pone.0237066 (PMC7418977; doi:10.1371/journal.pone.0237066)
Supplement: Attachment — Submitted filename: Response to Reviewers.docx [file pone.0237066.s003.docx]

**Answer to reviewers**

**Reviewer #1:**

**Major Concerns:**

***1) Why were female mice only used? Any role of the estrogen cycle?***

Thank you very much for you comment. We used female mice as previous results from our laboratory demonstrated that females are more sensitive to the neuroinflammatory effects of alcohol effects than males (Alfonso-Loeches et al., 2013; Pascual et al., 2017). A comment has been included on p. 5. Another reason was the price to perform next-generation sequencing (NGS). In fact we initially programmed to perform NGS in the cortices of males and females, but the prices to do NGS were twice as high as we had to add four additional groups (WT, WT + Ethanol, TLR4-KO, TLR4-KO +ethanol).

Regarding the effects of the estrogen cycle, although they are very important and could mediate some effects, we did not consider them in the present study.

**2) *Discussion is a bit long.***

As suggested, we have attempt to reduce the Discussion but, at the same time, we have included some comments in response to the reviewers, which made the Discussion longer.

***Minor Concerns*:**

***1) Please have a*** ***native English speaker edit for language.***

Thank you very much for your comment. The manuscript has been revised by a native English speaker.

**Reviewer #2: Comments to author.**

***1) The sample-size used for the NGS analysis is very small (******n=3 WT vs n=2 TLR4 KOs). This is a big concern as NGS technologies, as all other biotechnologies, are affected by both biological and technical variability. It would be beneficial to increase the n-number of the groups to gain statistical power and achieve comparability with your very clear qPCR data.***

Thank you very much for your comment. As stated in the Material and Methods section (p. 7), each sample is a pool from three different mice cortices and, therefore, the three *pools in the WT* is the average of nine different cortices. However in TLR4 KO, we had problems with one pooled sample and we used only two pooled samples that were the average of **six** mice cortices. We have modified the section RNA Quantity and quality determinations paragraph to make it clearer (p. 7-8).

***2) In your methods section, please provide details on brain extraction and dissection: Did you perfuse the brains? Did you use a brain matrix? What were your estimated coordinates and the thickness of the brain section?***

Thank you very much for your question. On p. 5 we have described details of the procedure used for brain extraction and cortices dissection, and how cortices were processed. Indeed we have added a new section ***Cerebral cortex dissection (p. 5)*** in which we describe the specific procedure used. Cortices dissection was performed by the same person using atlas coordinates. Cortices were weighed and immediately snap-frozen in liquid nitrogen until used for further determination analyses. The weight of cortices was similar in all the groups.

***3) It would be relevant to include in your discussion a comparison to the findings of your previous TLR4 study (Ureña-Peralta et al., 2018), as some of the miRNAs uncovered are similar****.*

*As suggested, we have discussed and compared previous findings to the present results. In particular, we have discussed the relevance of some miRNAs that were dysregulated in the ethanol-treated WT mice, but not in TLR4-KO mice, as lack of TLR4 receptors down-regulates these miRNAs pathways (p. 24).*

***4) Please discuss the limitations of the global TLR4 KO strategy: Are you expecting these effects to be due to CNS TLR4s or secondary effects due to*** ***peripheral*** ***immune-to-brain signalling,*** ***or*** ***blood-brain-barrier endothelial TLR4s?***

As suggested, we have discussed both the limitation of the TLR4 KO strategy to study neuroinflammation, and the potential involvement of peripheral immune-to-brain signaling in neuroinflammation (p. 24)

***General manuscript comments:***

***5) The image resolution of the figures is very poor! It in many cases not possible to read the figure labels. It makes it extremely difficult to evaluate consistency between what is written in the results-section and the actual data. Please revise.***

We appreciate your comment and have included the images in TIFF. We hope that they it is now easier to evaluate and observe the details and figure labels.

**6) *Several of the figure-legends are incomplete, for instance some are missing n-numbers, while the figure legend for figure 1 is unstructured and missing (C) and (D). Please revise the figure-legends.***

Thank you very much for drawing our attention, and we apologize for these mistakes. We have revised all the figures legends and included the correct numbers. Figure 1 has been arranged.

***7) The language quality varies throughout the individual sections of the manuscript. The NGS-methods and discussion-sections are very well written, while the introduction has lacks (see minor comments below). Please make the language more coherent.***

We thank the reviewer for drawing our attention *and have corrected the unclear text and sentences.*

***-Language, typos and understanding corrections:***

***-Please correct PAMPs from pattern associated molecular pattern to pathogen associated molecular pattern page 3 line 3.***

Thank you very much. We have corrected all the typos.

***-Please revise the sentence: “The sequence-complementary mechanism of miRNA activity exploits combinatorial regulate the expression of genes by repressing the translation of their complementary target genes (9). Page 3, section 3, line 2. Do you mean: (“…activity exploits combinatorial regulation of gene expression by…”?).***

These sentences have been revised and appropriately changed.

***-Please revise aim 2 on page 4, it is unclear what you mean: “2) if there are compensatory changes in certain miRNAs that could explain the lack of effects with the neuroinflammation associated with alcohol intake”.***

Thank you, they have been corrected.

***-In the introduction you refer to your previous study (ref 17), which clearly is linked to this aim, however you don’t specify what the “lack of effects” were.***

We have clarified *“lack of effects”.*

***-Furthermore, based on this study a question was raised “These results raise the question as to what the impact of miRNAs profile in mice is lacking TLR4 response?”. If this question is linked to aim 2, it needs to be further clarified, also it is unclear what you mean – please revise the question.***

The question in aim 2 has been changed to a more specific aim.

***-On page 5, under the Alcohol treatment section, line 10-11: You have written “obtained” several times; hence the sentence has become fragmented, also please state the actual BAL-values as this is useful information for the reader.***

The sentence has been rewritten. Thank you for drawing our attention to this.

***On page 10 under the statistical section, line 2, Western blotting results are mentioned. However, there is no description of such method or data elsewhere in the manuscript.***

On page 10, the sentence of the “Western blotting results” has been eliminated.

***On page 19, line 1: “Figure 4B, obtained from, illustrate the two subfamilies…”, please specify what it was*** ***“obtained from” or simply delete the obtained from, it is unclear what you mean.***

As suggested, we have deleted “obtained from”.

We would like to thank the reviewer for his/her help and suggestions to clarify some parts of the text. All the comments have been taken into account and corrections made.

Reviewer #3: Summary

**Critique**

***1) The manuscript is technically sound and the statistical analysis has been performed appropriately and rigorously. The results obtained by sequencing are always validated by RT-PCR and the appropriate controls are included in each figure. Moreover, the data support the conclusions.***

***2) One major concern is the observation that TLR4KO mice show depletion of TLR7/8. How can you be sure that the micro-RNA differential expression is not due to the lack of TLR7/8? Do TLR7/8 mice show micro-RNA-dysregulation?***

Thank you very much for your appropriate comment. We have performed an additional experiment to evaluate the gene expression levels of receptors TLR7 and TLR8, as well as two genes involved in this pathway. In Fig. 6 we now show that while ethanol increased the TLR8 mRNA levels in both genotypes, no changes in gene expression were noted in TLR7 compared to WT and TLR4KO (see Fig. 6, the results on p. 22 and the Discussion on p. 25 ).

.***3) The authors could focus on a few relevant*** ***micro-RNAs involved in inflammation that are differentially expressed and identify the effectors of the TLR4 signalling pathway that modulate these micro-RNAs.***

Thank you for your comment; the genes analysed in figure 1 (Il1R1, Traf6 and IL6) in addition to the INFα, analyzed in the new figure (Fig. 6), are related with the miR200 family. We hope you have resolved your doubts.

***4) The authors could then exogenously modulate the expression (overexpression or knockdown) or function of specific effectors in TLR4 KO mice to rescue the expression of specific micro-RNAs.***

We appreciate your comment. In fact although we have attempted to exogenously modulate the expression of TLR4-KO in cortical glial cells in culture from the TLR4-KO mice, we still obtained no clear conclusions.

***5) While the statistical analysis has been performed appropriately, the description of the statistical analysis should be described for each panel (it is for example missing in Figure 3b).***

We appreciate your comment and have included the statistical analysis in all the figure legends.

***6). Figure resolution is low and figure layout can be improved. In Figure 4, for example, some panels are extremely big (Figure 4a) while others are barely visible (Figure 4b).***

Thank you very much for your comment. We have provided figures at higher resolutions. The panels for Fig. 4A and 4B have been modified.

***7). Figure 1: Figure 1C-D description is missing in the figure legend.***

We apologize for this error. We have modified the legend of Figure 1.

***8). Figure 4b: Specify that the chromosomal localization refers to the human genome both in the legend and the main text, where there is only reference to the mouse genome, which could create confusion.***

Thank you very much for your useful comment. We have now modified the text according to your suggestions.
